# The Effect of Depression on Serum VEGF Level in Alzheimer's Disease

**DOI:** 10.1155/2015/742612

**Published:** 2015-03-08

**Authors:** JaeHoon Jung, SoYeon Kim, KyungHee Yoon, YooSun Moon, Daeyoung Roh, SangKyu Lee, KyungChan Choi, JunSub Jung, DoHoon Kim

**Affiliations:** ^1^Department of Psychiatry, Chuncheon Sacred Heart Hospital, Hallym University College of Medicine, Chuncheon 200-704, Republic of Korea; ^2^Institute for Skeletal Aging, Chuncheon Sacred Heart Hospital, Hallym University College of Medicine, Chuncheon 200-704, Republic of Korea; ^3^Department of Pathology, Chuncheon Sacred Heart Hospital, Hallym University College of Medicine, Chuncheon 200-704, Republic of Korea; ^4^Department of Pharmacology, College of Medicine, Institute of Natural Medicine, Hallym University, Chuncheon 200-704, Republic of Korea

## Abstract

*Objective*. Growing evidence suggests that angiogenesis might represent a new pathogenic mechanism involved in the progression of Alzheimer's disease (AD). Among angiogenic cytokines, vascular endothelial growth factor (VEGF) levels in AD patients have been evaluated, but the results are controversial among studies. We investigated serum levels of VEGF in AD patients with depression, AD patients without depression, and the controls, respectively. The aim of this study is to elucidate the relationship between VEGF, depression, and cognitive impairment in AD. *Methods*. The CDR (Clinical Dementia Rating), MMSE-KC (the Mini-Mental Status Examination-Korean version), and SGDS-K (the Korean version of the Geriatric Depression Scale-Short Form) were measured in the subjects. Serum VEGF levels were measured in 24 AD patients with depression, 25 AD patients without depression, and 26 controls, using an enzyme-linked immunosorbent assay kit. *Results*. Serum VEGF levels in AD patients with depression were significantly higher than AD patients without depression or the control. A correlation was observed between VEGF and scores on SGDS-K, but no correlation was detected between VEGF and MMSE-KC scores. *Conclusion*. Serum VEGF levels in AD patients with depression were higher than those without depression. Depression might be associated with changes in serum levels of VEGF in AD patients.

## 1. Introduction

Although the most widely convincing hypothesis for the etiology of Alzheimer's disease (AD) is the amyloid cascade hypothesis [1], growing evidence suggests that angiogenesis might represent a new pathogenic mechanism involved in the progression of AD [2]. Among angiogenic cytokines, vascular endothelial growth factor (VEGF) has recently received attention. VEGF was originally known as a growth factor playing a part in vascular permeability and migration of endothelial cells [3]. VEGF expression is often induced by brain hypoxia [4] or upregulated by chronic inflammation [5]. As a potential mediator of angiogenesis, VEGF is present in the walls of intraparenchymal vessels in the brains of AD patients, in diffuse periventricular deposits, and in clusters of reactive astrocytes [6]. The increased reactivity in VEGF might implicate the presence of regulatory mechanisms compensating for insufficient vascularity and reduced cerebral perfusion in AD [7]. It is suggested that angiogenic activation of the brain endothelium in AD leads to deposition of the beta amyloid plaque and secretion of a neurotoxic peptide that kills cortical neurons [8]. Thus, it can be speculated that abnormal regulation of VEGF might be involved in the process of cognitive impairment in AD [8, 9].

Several studies have measured VEGF levels in AD patients, but the VEGF levels were not consistent in AD patients. Lower concentrations of VEGF have been detected in the serum of AD patients [10], whereas other reports showed that VEGF levels in AD patients were higher than those of controls [9, 11].

A recent review shows higher prevalence rates of depressive symptoms in AD patients approximately range from 20% to 30% [12]. AD is often associated with depressive symptoms developing at any time before or after onset of AD [13]. The interrelationship between depression and dementia is complex and not well understood. It is clear that chronic inflammation plays a role in neurodegenerative changes in depression and AD [14]. Many chronic inflammatory markers, such as cytokines and growth factors, have been investigated as possible mediators linking depression and dementia.

Although the possible role of VEGF in depression has been hypothesized in the context of the neurotrophic model of depression, it has become evident that there are conflicting data regarding VEGF levels in patients with depression [15]. To our knowledge, no studies have measured serum VEGF levels in AD patients with comorbid depression. VEGF levels were measured in serum of AD patients with and without depression in this study. The aim of this study was to compare serum VEGF levels in AD patients with depression (AD + depression) with those in AD patients without depression (AD − depression). Moreover, we analyzed the association of VEGF levels with depression and cognitive impairment in AD patients.

## 2. Methods

### 2.1. Study Participants and Procedures

The subjects consisted of 24 Korean patients with AD + depression, 25 patients with AD − depression, and 26 healthy controls over 65 years of age. For determining sample size, we suppose that the group VEGF difference and standard deviation (*α* = 0.025, *β* = 0.2) are 60 and 80, respectively (for review, see [15]). Thus, sample size would be about 27.

This study protocol was approved by the Ethics and Medical Research Committee of Chuncheon Sacred Heart Hospital (IRB protocol number 2011-66), and written informed consent was obtained from all participants. Patients who visited the Dementia Clinic of Hallym University Medical Center and signed informed consent were evaluated for dementia and depression.

Subjects did not take any medicine except antihypertensive agents and were excluded if they had an inflammatory disease, malignancy, vascular dementia, other neurodegenerative diseases diagnosed after a full neurological and medical examination, diabetes mellitus, atherosclerosis, fracture, or substance abuse or were taking any medicines known to influence serum angiogenic factors (such as acetylsalicylic acid or 5 alpha-reductase inhibitors). Antihypertensive medicines were taken in AD + depression (*n* = 16), AD − depression (*n* = 14), and controls (*n* = 9) (Table 1). Their current smoking status was questioned and their height and body weight were measured to calculate BMI (body mass index).

### 2.2. Measures

#### 2.2.1. Diagnosis

Each subject was examined by a board-certified psychiatrist trained in the survey methods and scales used in this study. The Korean version of the Consortium to Establish a Registry of Alzheimer's Disease consists of a standardized clinical interview of demographic information, cognitive and functional status, drug inventory, depression and medical history, and general physical and neurological examinations [16]. The Clinical Dementia Rating (CDR) [17] and Mini-Mental Status Examination-Korean version (MMSE-KC) were used [18]. Scores on the MMSE-KC range from 0 to 30, with higher scores indicating better cognitive function. The global CDR is established by clinical scoring rules where CDR 0 indicates no dementia and CDR 0.5, 1, 2, or 3 indicates questionable, mild, moderate, or severe dementia, respectively.

The diagnosis of AD with depression was based on Provisional Diagnostic Criteria for Depression of Alzheimer's Disease [19]. These criteria have been recently developed by a working group of the National Institute of Mental Health, who modified the DSM-IV-TR criteria for the diagnosis of mood disorder in an attempt to identify the presence of depression in AD more precisely than the previous DSM-IV criteria [19–21].

The severity of depressive symptoms was measured using the Korean version of the Geriatric Depression Scale-Short Form (SGDS-K) [22]. The SGDS-K is composed of 15 items with similar capability of measurement compared to the 30 items on the Geriatric Depression Scale. This scale involves 15 items scored with “yes” or “no” responses about depressive symptoms, and scores range from 0 to 15, a higher score indicating more severe depressed status.

#### 2.2.2. Serum VEGF

Nonfasting blood samples were drawn in a sitting position between 9 AM and 4 PM from an antecubital vein, stored in plain tubes without EDTA, and centrifuged at 1610 g for 10 min at 4°C. The sampling procedures were performed by two well trained medical personnel in a standardized manner. The sample had been collected for 1 year. The serum samples were stored at –80°C until use at most for 1 year. Serum levels of VEGF were measured with an enzyme-linked immunosorbent assay kit (VEGF; R&D Systems, Minneapolis, MN, USA). The measurements were done in duplicate on 96-well plate. Serum VEGF determinations with an intra-assay variance below 10% were included in the study (CV max; nearly 9%). Three internal VEGF controls (low: 101–191 pg/mL, medium: 321–591 pg/mL, and high: 667–1221 pg/mL), commercially available (R&D Systems, Minneapolis, MN, USA), were included on each plate.

#### 2.2.3. Statistical Analysis

The statistical analysis was performed using the Statistical Package for the Social Science ver. 18.0 program (SPSS, Inc., Chicago, IL, USA). Pearson's chi-square was used to test the categorical variables. Quantitative and qualitative variables were described through means ± standard deviations (SDs). One-way analysis of variance was conducted to evaluate group differences between AD + depression, AD − depression, and control participants. Duncan's test was used for the post hoc analysis. Correlations between variables were tested using Pearson's correlation coefficient. A *P* value < 0.05 was considered significant.

## 3. Results

### 3.1. Demographic Data

The demographic characteristics of the study population are summarized in Table 1. The mean ages of the AD + depression, AD − depression, and control subjects were 77.00 years (SD = 8.02; range 66–106 years), 76.88 years (SD = 5.56; range 66–84 years), and 74.08 years (SD = 4.15; range 68–82 years), respectively.

No significant differences among AD + depression, AD − depression, and controls were observed in age, sex distribution, body mass index, and hypertension.

The average SGDS-K score for AD + depression was 9.96 (SD = 3.58, range 3–15), that for AD − depression was 2.50 (SD = 2.58, range 0–9), and that for control subjects was 0.58 (SD = 0.86, range 0–3), indicating significantly higher levels in AD + depression than in AD − depression or the controls (*F*(2,69) = 91.44, *P* = 0.000). The average MMSE-KC score for AD + depression was 15.88 (SD = 3.58, range 8–22), that for AD − depression was 14.72 (SD = 3.71, range 8–22), and that for control subjects was 27.77 (SD = 1.63, range 25–30) (*F*(2,72) = 138.51, *P* = 0.000). The average CDR score for AD + depression was 0.96 (SD = 0.39, range 1-2) and that for AD − depression was 1.08 (SD = 0.69, range 1–3) (*F*(1,47) = 0.576, *P* = 0.452).

### 3.2. Serum VEGF Levels

**Figure 1:**
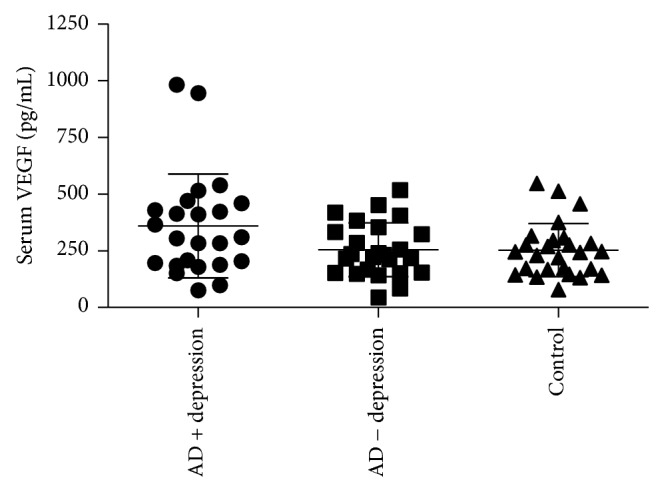
Serum vascular endothelial growth factor (VEGF) levels in AD + depression (*n* = 24), in AD − depression (*n* = 25), and in control subjects (*n* = 26). The AD + depression had higher serum VEGF levels compared to those of AD − depression or controls (*F*(2,72) = 3.51, *P* = 0.035). Bars indicate standard deviation.

Serum VEGF levels in each group are shown in Figure 1. Serum VEGF levels in AD + depression, AD − depression, and control subjects were 254.02 ± 118.75, 359.24 ± 228.33, and 252.40 ± 117.43 pg/mL (mean ± SD).

In analysis with ANOVA with post hoc test, the AD + depression had higher serum VEGF levels compared to those of AD − depression or controls (*F*(2,72) = 3.51, *P* = 0.035). Additionally, a univariate analysis of covariance (ANCOVAs) was conducted for each group's differences in serum levels of VEGF, controlling for sex, BMI, and hypertension. Serum VEGF levels in AD + depression were significantly higher than AD − depression or control (*F* = 4.834, *P* = 0.011).

### 3.3. Correlation between Serum VEGF Levels with Depression and the Cognitive Function Scales

A positive correlation was observed between serum levels of vascular endothelial growth factor (VEGF) and SGDS-K scores (*r* = 0.24, *P* = 0.04). But significant correlation was not observed within the AD + depression group (*r* = −0.06, *P* = 0.78). No significant correlation was observed between serum VEGF and the MMSE-KC scores (*r* = −0.083, *P* = 0.48). No significant correlation was observed between serum VEGF and the CDR scores (*r* = 0.18, *P* = 0.22).

## 4. Discussion

This study explored the hypothesis that VEGF may be possible mediator linking depression and dementia and might be involved in the cognitive impairment in AD.

The result showed that serum VEGF levels in AD + depression were higher than those in AD − depression. This might be the first study to detect serum VEGF levels in AD + depression. Our results demonstrated that serum VEGF levels were positively correlated with the SGDS-K scores but not with the MMSE-KC scores. This finding suggests that serum VEGF levels might be associated with depressive symptoms in AD patients.

Although it has been suggested that VEGF might play an important role in pathogenesis of AD, VEGF levels in AD patient have been controversial. One study showed the elevated intrathecal VEGF levels in patients with AD [9] and another study found that VEGF serum levels were higher in patients with AD than controls [11]. Significantly increased levels of VEGF of the hippocampal cortex of AD patients, compared with normal brain, were reported recently [23]. Lower serum VEGF concentrations were found in patients with AD [10]. A significant decrease in VEGF levels was found in AD patients, compared to the healthy old and vascular dementia groups [24]. In this study, serum VEGF levels were higher in AD + depression than in AD − depression. According to our results, the presence of depression might influence serum levels of VEGF. Therefore, studies measuring neurotrophic function of serum VEGF levels in AD patients should evaluate depressive symptoms to minimize the effect of the possible coexistence of both diseases.

Several studies have reported increased VEGF levels in various human tissues of depressive patients. It was reported that depression, severity of depression, and previous depressive episodes are associated with higher serum VEGF levels [25]. A significant increase in plasma VEGF levels in patients with MDD compared to those in control subjects was reported [26]. Plasma VEGF levels of patients with acute episodes of MDD and bipolar disorder are significantly higher than those in controls [27].

The mechanism of how VEGF levels are increased in patients with depression is unclear. One of the possible mechanisms is that elevated serum VEGF levels might be induced by psychosocial stress. It is well known that there exist associations between psychosocial stressors and depression [28, 29]. VEGF can be derived from increased norepinephrine through beta-adrenergic receptors, activated by sympathetic pathways [30, 31]. The significantly incresed level of plasma VEGF in women exposed to prolonged psychosocial stress was found [32]. Serum VEGF levels were increased in patients with cancer under higher psychosocial stress than those with lower stress [33]. Increased serum VEGF levels could be explained by norepinephrine that can be induced by stress. Thus, increased serum VEGF levels in patients with AD with depression might suggest that AD patients have higher psychosocial stress related to depression, which might induce activation of sympathetic nervous system. Further studies are needed to validate these relationships. Another possible explanation is that elevated serum VEGF levels might be induced by compensatory mechanism. A neuroprotective or neurotrophic role of VEGF in patients with depression has been suggested in some studies [34, 35]. Neuronal neogenesis in the brain is deprived due to stress related to depression, and paradoxically, serum VEGF might be increased to compensate this deprivation [27, 35, 36].

In our study, a significant difference in serum VEGF levels was observed between depressed and nondepressed counterparts in AD. The serum VEGF levels were not related with cognitive impairment, but with depressive symptoms. Serum VEGF was previously suggested to be a useful biomarker for depression [15]. Our findings provided further support for this finding, at least in elderly people with neurodegenerative changes. Thus, our results assume that the increase of serum VEGF levels may suggest the presence of depressive symptoms in AD. However, several recent studies have reported the relationship between serum VEGF levels and smoking [37, 38], fat mass [37, 39], and gender [37, 38]. Additionally, various drugs and diseases should be checked because such factors may affect the serum level of VEGF. In our study, study subjects did not take any medicine except antihypertensive agents and were excluded if they had various diseases or were taking any medicines known to influence serum angiogenic factors. In this study, serum VEGF levels were not correlated with MMSE-KC scores. It is speculated that abnormal regulation of VEGF might be involved in the process of cognitive impairment in AD through activation of deposition of neurotoxic agent such as the beta amyloid plaque [8, 40]. Our study was a cross-sectional study and included only mild to moderate AD. Thus, a prospective study may be needed to explore the relationship between serum VEGF levels, cognitive function, and CSF beta amyloid level. In our study, although a significant positive correlation was observed between depressive symptoms and VEGF in the whole group (*r* = 0.25, *P* = 0.04), no significant correlation was observed within the AD + depression group (*r* = −0.06, *P* = 0.78). In the primary analysis, the AD + depression had higher serum VEGF levels compared to those of AD − depression or controls. It could be that depression may influence serum levels of VEGF, but VEGF might not be a biomarker representing a degree of severity of depression in AD.

The following are limitations of this study. The sample number was relatively small. Further study may be needed with a larger sample size for generalization of these results. Because this study was a cross-sectional study, we measured serum VEGF levels at the same time in AD patients with depression and a longitudinal study is needed to compare VEGF levels with improvements in depressive symptoms to demonstrate the effectiveness of VEGF as a prognostic factor. In the present study, we cannot fully determine specific interaction between depression and AD. Although serum VEGF levels were not elevated in AD patients without depression, AD might be predisposed to increase serum VEGF levels in AD patients with depression. The inclusion of depressed elderly people without comorbid AD might be helpful to verify this. Finally, the imaging studies were not performed in this study. Recent studies have reported that patients with geriatric depression showed more white matter hyperintensity (WMH) in the brain, which are related with cerebrovascular diseases [41, 42]. Therefore, when AD patients have comorbid depression, they could have a higher probability of having WMH and cerebrovascular diseases. These factors might influence the serum VEGF levels. Further evaluations verifying the relationship between WMH and VEGF levels in AD patients would be needed in the future.

## 5. Conclusion

The study aim was to investigate the association between VEGF and depression and cognition in Alzheimer's patients. Increases in serum VEGF levels were observed in patients with AD and comorbid depression. Serum VEGF levels were correlated with depressive symptoms, but no correlation was detected between VEGF and cognition.

These results suggest that the pathophysiology and progression of AD might be altered if depression occurs with AD. The presence of depression in AD patients should be suspected when vascular factors are altered as one of trait markers of depression in AD. VEGF could be a possible mediator in the neurobiological mechanism of action linking AD and depression.

## Figures and Tables

**Table 1:** Demographic and clinical characteristics of study subjects.

Variables	AD + depression (*n* = 24)	AD − depression (*n* = 25)	Control (*n* = 26)	Statistics	*P*
	Variables	Mean ± SD	Mean ± SD		
Sex (M/F)	4/20	5/20	11/15	*χ* ^2^ = 5.05	0.080
Age	77.00 ± 8.02	76.88 ± 5.56	74.08 ± 4.15	*F* = 1.89	0.158
Hypertension (%)	14 (58.33%)	16 (64%)	9 (34.62%)	*χ* ^2^ = 4.98	0.083
BMI	22.59 ± 2.56	21.93 ± 2.74	23.34 ± 3.04	*F* = 1.65	0.200
Smoking	4 (16.67%)	2 (8%)	1 (3.85%)	*χ* ^2^ = 2.50	0.286
SGDS-K	9.96 ± 3.58	2.50 ± 2.58	0.58 ± 0.86	*F* = 91.44	0.000
MMSE-KC	15.88 ± 3.58	14.72 ± 3.71	27.77 ± 1.63	*F* = 138.51	0.000
CDR	0.96 ± 0.39	1.08 ± 0.69	0 ± 0	*F* = 0.58	0.452

Analysis of variance was used to determine the *P* value compared to the control. *Note*. AD + depression, Alzheimer's disease with depression; AD − depression, Alzheimer's disease without depression; Control, normal control; SD, standard deviation; BMI, body mass index; MMSE-KC, Mini-Mental State Examination-Korean version; SGDS-K, Korean version of the Geriatric Depression Scale-Short Form; CDR, Clinical Dementia Rating scale.

## Abbreviations

AD: Alzheimer's disease
VEGF: Vascular endothelial growth factor
CDR: Clinical Dementia Rating
MMSE-KC: Mini-Mental Status Examination-Korean version
MDD: Major depressive disorder
SGDS-K: Korean version of the Geriatric Depression Scale-Short Form
SD: Standard deviation.
